# Impact of Elemental Sulfur on the Rhizospheric Bacteria of Durum Wheat Crop Cultivated on a Calcareous Soil

**DOI:** 10.3390/plants8100379

**Published:** 2019-09-27

**Authors:** Dimitris L. Bouranis, Anastasia Venieraki, Styliani N. Chorianopoulou, Panagiotis Katinakis

**Affiliations:** 1 Plant Physiology and Morphology Laboratory, Crop Science Department, Agricultural University of Athens, Iera Odos 75, 118 55 Athens, Greece; s.chorianopoulou@aua.gr; 2 General and Agricultural Microbiology Laboratory, Crop Science Department, Agricultural University of Athens, Iera Odos 75, 118 55 Athens, Greece; venieraki@aua.gr

**Keywords:** elemental sulfur, calcareous soil, arylsulfatase-producing bacteria, P mobilization, Fe mobilization, nutritional fortification

## Abstract

Previous experiments have shown that the application of fertilizer granules containing elemental sulfur (S^0^) as an ingredient (FBS^0^) in durum wheat crops produced a higher yield than that produced by conventional ones (F), provided that the soils of the experimental fields (F vs. FBS^0^) were of comparable quality and with the Olsen P content of the field’s soil above 8 mg kg^−1^. In this experiment the FBS^0^ treatment took place in soil with Olsen P at 7.8 mg kg^−1^, compared with the F treatment’s soil with Olsen P of 16.8 mg kg^−1^, aiming at reducing the imbalance in soil quality. To assess and evaluate the effect of FBS^0^ on the dynamics of the rhizospheric bacteria in relation to F, rhizospheric soil at various developmental stages of the crops was collected. The agronomic profile of the rhizospheric cultivable bacteria was characterized and monitored, in connection with the dynamics of phosphorus, iron, organic sulfur, and organic nitrogen, in both the rhizosoil and the aerial part of the plant during development. Both crops were characterized by a comparable dry mass accumulation per plant throughout development, while the yield of the FBS^0^ crop was 3.4% less compared to the F crop’s one. The FBS^0^ crop’s aerial part showed a transient higher P and Fe concentration, while its organic N and S concentrations followed the pattern of the F crop. The incorporation of S^0^ into the conventional fertilizer increased the percentage of arylsulfatase (ARS)-producing bacteria in the total bacterial population, suggesting an enhanced release of sulfate from the soil’s organic S pool, which the plant could readily utilize. The proportion of identified ARS-producing bacteria possessing these traits exhibited a maximum value before and after topdressing. Phylogenetic analysis of the 68 isolated ARS-producing bacterial strains revealed that the majority of the isolates belonged to the *Pseudomonas* genus. A large fraction also possessed phosphate solubilization, and/or siderophore production, and/or ureolytic traits, thus improving the crop’s P, Fe, S, and N balance. The aforementioned findings imply that the used FBS^0^ substantially improved the quality of the rhizosoil at the available phosphorus limiting level by modulating the abundance of the bacterial communities in the rhizosphere and effectively enhancing the microbially mediated nutrient mobilization towards improved plant nutritional dynamics.

## 1. Introduction

Plant nutrient acquisition from the soil via the root system is influenced by a wide range of factors. Plant species and genotype, the soil type and chemical‒physical characteristics, the soil microorganism communities, the fertilization regime, and environmental conditions are all factors of central agronomic importance. In this context, the biological activities of microorganisms can play an important role [1]. Regardless of the plant species, a fraction of the soil microbiome will eventually colonize the soil bordering the root surface (i.e., the rhizosphere), because plant roots release a wide range of compounds involved in attracting microorganisms, which may be beneficial, neutral, or detrimental to plants [2]. Therefore, a zone of intense microbial activity is generated that can affect plant nutrient acquisition processes by influencing nutrient availability in the rhizosphere and/or the plant biochemical mechanisms underlying the nutritional process [3], thus profoundly influencing crop fortification and production [4]. The beneficial bacteria residing in the rhizosphere, referred to as plant growth-promoting rhizobacteria (PGPR), are in general capable of enhancing the growth of plants and/or protecting them from biotic or abiotic stresses [5]. The PGPR that are involved in soil nutrients’ mobilization, thus enhancing the availability of the mobilized nutrients to the plant, are usually endowed with traits that stimulate the mobilization of unavailable plant nutrients through various mechanisms, including phosphate solubilization and production of siderophores, among several others. 

Phosphate mineralization by microorganisms involves either acidification of the extracellular environment by production of organic acid anions, such as gluconic acid, (acid or alkaline) phosphatase activity, or phytase activity. The use of P-solubilizing microorganisms has received much attention over the last few decades as a means of mobilizing P in different types of soils and fertilization regimes. Given that micronutrient deficiency generally occurs in calcareous soils because of high pH, acidification of the rhizosphere can increase the solubility of micronutrients [1,6,7,8,9,10,11,12]. 

A large fraction of the agricultural soils in the Mediterranean area show an alkaline pH, thus the bioavailable Fe fraction is lower than that required for optimal plant growth. PGPR might increase the bioavailability of insoluble Fe by the production and secretion of siderophores, rendering it available for both bacteria and plants. There is a considerable body of evidence showing that several siderophore-producing microorganisms are able to enhance Fe uptake in monocot and dicot plants species [4,13,14,15,16]. 

The role of bacteria capable of mobilizing S from the soil organic matter, thus providing S available to plants, has attracted little attention so far, despite the fact that most of the S in soils is bound to organic molecules, making up more than 90% of the S present in soils [17,18,19,20]. Evidence has been provided that plant S fortification of *Miscanthus giganthus* is correlated with the population density of rhizobacteria that transform organic sulfur into bioavailable forms [21]. Furthermore, it has been demonstrated that S-demanding plants, such as rape, appear to recruit higher numbers of arylsulfatase (ARS)-producing bacteria as compared to barley [22]. Arylsulfatase hydrolyzes sulfate esters to sulfate and is a key enzyme of soil organic S mineralization, so is often utilized as a soil fertility indicator [23,24,25]. 

The application of fertilizer granules with incorporated elemental sulfur (FBS^0^) in durum wheat crop produced a higher yield and higher S and Fe contents compared with the application of the conventional (F) ones, provided that the experimental fields (F and FBS^0^) were of comparable quality. The fertilization with FBS^0^ at sowing mobilized iron from the rhizosoil, thus providing more iron to the crop; in addition, it boosted the crop’s sulfur content [26]. On the other hand, the level of available phosphorus was found to be correlated with the corresponding relative change in the yields (YFBS^0^/YF), presenting a strong positive relationship, and the content of 8 mg kg^−1^ was a turning point; lower values resulted in a lower yield compared with the conventional crop [27]. 

In this experimental approach, the FBS^0^ treatment took place in soil of inferior quality and with an Olsen *p* value of 7.8 mg kg^−1^, compared to the F treatment’s soil with adequate quality and an Olsen P of 16.8 mg kg^−1^. The working hypothesis was that the FBS^0^ treatment could possibly alleviate the anticipated yield loss produced by the control treatment at an Olsen P of 7.8 mg kg^−1^. Moreover, it was hypothesized that the small amount (2% w/w) of S^0^ added in with the fertilizer application at sowing stimulated the recruitment of rhizobacteria that are able to mobilize unavailable P, Fe, and S into bioavailable forms. Thus, the aims of this study were (i) to monitor the effect of elemental sulfur as an ingredient of the applied fertilization scheme on phosphorus and iron dynamics in the soil explored by the root system mass, i.e., the rhizosoil (RS), as well as in the aerial part of the plant during development under the specific regimes, i.e., when the FBS^0^ treatment takes place in soil with available phosphorus at a turning point level of 8 mg kg^−1^, compared to the level of 16 mg kg^−1^ of the F treatment’s soil; and, in parallel, (ii) to monitor the dynamics of rhizospheric bacteria capable of transforming unavailable P, Fe, and S into bioavailable forms, under the circumstances, in order to elaborate on the effect of the small added amount of S^0^ on the crop’s rhizospheric microbiome, if any, under the two aforementioned levels (marginal vs. adequate) of available phosphorus.

## 2. Materials and Methods

### 2.1. Experimental Setup

**Figure 1:**
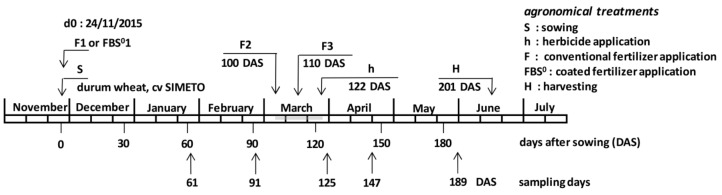
Overview of the experiment. The gray line in March shows the extent of the agronomical treatments. Phenological phases at each sampling day: 61 DAS, tillering; 91 and 125 DAS, stem elongation; 188 DAS, grain filling. Heading was apparent at 133 DAS.

Durum wheat (*Triticum turgidum* L. *subsp. durum* (Desf.) Husn.), commercial cultivar SIMETO, was sown at Arma (latitude 38.35° N, longitude 23.49° E, 256 m asl) in Viotia county, central Greece, in a production area of 1.8 ha with calcareous soil. This area was chosen due to its heterogeneity in Olsen P content and was divided into two experimental fields of 0.9 ha each. The field with an Olsen *p* value of 16.8 mg kg^−1^ was subject to conventional F treatment according to the local agricultural practices (F crop), while the other one, with an Olsen *p* value of 7.8 mg kg^−1^, received the corresponding FBS^0^ treatment (FBS^0^ crop, Figure 1). Sowing and fertilizer application took place on 24 November 2015 (day 0). The control crop was fertilized with a commercial 16-20-0 fertilizer (nitrogen was provided as ammonium sulfate, phosphorus as triple superphosphate) at a rate of 275 kg ha^−1^. The FBS^0^-treated crop received 280.5 kg ha^−1^ by applying the corresponding FBS^0^ 16-20-0 commercial fertilizer carrying 2% S^0^ (F: 275; S^0^: 5.5). At 100 and 110 days after sowing (DAS), additional fertilization (topdressing) with a commercial 40-0-0 fertilizer (urea plus ammonium sulfate; UAS) took place at the rate of 180 kg ha^−1^, according to the local practice and the same for both treatments. At 122 DAS, herbicide application took place at the rate of 500 mL ha^−1^ (Pasifica Bayer CropScience Ltd, Cambridge, UK).

Within each experimental field, the soil quality was tested prior to sowing by analyzing a representative composite sample collected at a depth of 20 cm. Then, an internal perimeter 7 m from the border was established and excluded from sampling and the internal area was divided into five groups. On each sampling day, at least five plants were collected from each group, with their root system and the surrounding soil by means of a shovel. The excess of soil was removed by hand and the soil explored by the root system mass, i.e., the rhizosoil (RS), was collected and analyzed. Afterward, the rhizosphere (Rhs) was collected from the root system by means of a brash. RS was monitored for phosphorus and iron concentration, while Rhs was used for microbiological procedures. In the dry mass (DM) of the aboveground plant part, the fluctuation dynamics of organic nitrogen, organic sulfur, phosphorus, and iron were monitored during the crops’ development. Thus, on each sampling day one composite sample of DM, RS, and Rhs per group was constructed and analyzed.

### 2.2. Determinations of Soil Parameters

Determinations of pH, organic matter, CaCO_3_, NO_3_^−^, Olsen P, exchangeable potassium, Fe-DTPA (DTPA: diethylenetriaminepentaacetic acid), Mn-DTPA, Cu-DTPA, and Zn-DTPA in the RS were performed according to the procedures described by Jones (1999) [28].

### 2.3. Determinations of Dry Matter, P, Fe, Organic N, and Organic S Concentrations in the Aerial Part of the Plant 

Samples of the aerial part of the plants were oven-dried at 80 °C and the dry mass was weighed and recorded. Then, it was ground to pass through a 40-mesh screen using an analytical mill (IKA, model A10, IKA^®^-Werke GmbH & Co. KG, Staufen, Germany). Prior to Fe and P analysis, samples were digested with hot H_2_SO_4_ and repeated additions of 30% H_2_O_2_ until the digestion was complete [28]. In the diluted dry mass (DM) digests, Fe content was determined by atomic absorption spectrophotometry (GBC, Model Avanta spectrophotometer, GBC Scientific Equipment PTY Ltd., Dandenong, Australia), while the P content was determined using the ammonium molybdate and stannous chloride procedure. Organic nitrogen (N_org_) was determined by micro-Kjeldahl digestion followed by distillation. The sulfate concentration was determined by extracting the oven-dried samples with 2% (*v*/*v*) acetic acid aqueous solution and by analyzing by a turbidimetric method [29,30]. Total sulfur content was determined after dry ashing at 600 °C [31]. The ash was dissolved in a 2% (*v*/*v*) acetic acid aqueous solution and filtered through Whatman No. 42 paper, and the total sulfate was determined turbidimetrically [29,30]. The content of organic sulfur (S_org_) was calculated by subtracting the total sulfate content from the total sulfur.

### 2.4. Microorganism Sampling, Isolation, Culture Conditions, and Arylsulfatase Activity

Immediately after collection, Rhs was placed on ice for further analysis. Rhs (3 g) was suspended in 30 mL of sterile phosphate-buffered saline (PBS), pH 7.2 on an orbital shaker (120 rev min^−1^) for 45 min. Cultivable bacteria were quantified by plating a serial dilution (three replicates, 10^−4^, 10^−5^, and 10^−6^) of soil suspension dilutions on nutrient agar medium, M9 mineral medium, and modified M9 mineral medium. The chromogenic arylsulfatase substrate 5-bromo-4-chloro-3-indolyl sulfate (X-Sulf, Sigma) was incorporated (100 mg L^−1^) into a modified M9 medium, as the sole sulfur source [24]. Plates were incubated at 28 °C for 48–72 h and colony-forming units were counted and expressed as cfu g^−1^ of soil. Microbial colonies possessing ARS activity were detected by their blue color on Petri dishes. Individual X-Sulf-utilizing strains were identified for further study from the highest dilutions showing growth in the modified M9 mineral medium. Single colonies were picked, re-cultured in modified M9 mineral medium, and maintained as glycerol stock (20%) at −80°C for further use.

### 2.5. Assessment of Plant Growth-Promoting (PGP) Traits

Apart from the arylsulfatase activity of the selected strains, we investigated other possible PGP traits, such as phosphate solubilization, siderophore production, urea solubilization, their swarming motility, and biocontrol activity. The phosphate-solubilizing activity of the isolates was studied on Pikovskaya agar [32]. Phosphate-solubilizing bacterial strains were identified by clear halo zones around their colonies. Sterile medium served as a control. Siderophore production was examined on chrome azurol-S agar medium [33]. CAS plates were spot-inoculated with a liquid culture of bacterial strains and observed for the development of an orange halo against a dark blue background around the colonies after 48 h of incubation at 28 °C. A change in color from blue to orange (hydroxamate-type siderophore) was considered a positive reaction. Urease production ability was tested by inoculating Urea Base Christensen ISO 6579, ISO 19250 (Conda, Madrid, Spain) medium Petri dishes. Phenol red indicator detects the alkalinity generated by the visible color change from orange to pink. Fluorescent pigment production was tested on King medium B and inspected under UV light. Bacterial strain swarming motility was tested by the inoculation of fresh swarm plates (nutrient agar, 0.5%). Plates were spotted with 1–5 µL of overnight broth culture of each strain, followed by overnight incubation (≤20 h) at 28 °C. Control plates were prepared in parallel with swarming ones with nutrient agar, 1.5% [34]. The bacterial isolates were also evaluated for their antagonistic potential against the soil-borne phytopathogenic fungi *Rhizoctonia solani* and *Fusarium oxysporum* [34]. Briefly, 5 μL drops of each bacterial culture (10^8^ cfu mL^−f^) were streaked equidistantly on the margins of NA plates. A mycelial agar plug of 5 mm diameter from a seven-day-old culture of *R. solani* and *F. oxysporum* grown on a PDA plate was placed in the center of the plate, between the two parallel streaks of the test bacterial isolate. Control plates were also prepared without the mycelial fragment. Two independent experiments were performed with each bacterial isolate. Plates were incubated at 25 °C for 7–10 days. All assays were done in triplicate.

### 2.6. PCR Amplification 16S rRNA Gene Sequence and Phylogenetic Analysis

Genomic DNA from bacterial cultures of the isolates was extracted using the NucleoSpin^®^ Microbial DNA Kit (Macherey-Nagel GmbH & Co KG, Düren, Germany) according to the manufacturer’s instructions. The DNA concentration and purity were assessed by a Nanodrop ND-1000 spectrophotometer (Thermo Fisher Scientific, Waltham, MA, USA). The primer set fD1 (5′-AGAGTTTGATCCTGGCTCAG-3′) and rP2 (5′-ACGGCTACCTTGTTACGACTT-3′) [35], targeting ribosomal DNA of approximately 1500 bp, was used for PCR amplification of the 16S ribosomal DNA. The polymerase chain reaction (PCR) amplification mixture contained 0.5 U KAPA2G Robust DNA polymerase (Kapa Biosystems Inc., Wilmington, MA, USA); 5 × KAPA2G GC buffer; 50 pmol of each oligonucleotide; and 50 ng of DNA template. A final volume of 50 μL was adjusted with distilled water. PCR reactions were cycled in a BIORAD thermocycler (MJ mini) with a hot start step at 94 °C for 5 min, followed by 35 cycles of 94 °C for 1 min, annealing at the appropriate temperature for 1.5 min, and 72 °C for 1 min, with a final extension step at 72 °C for 10 min. The 16S rRNA gene sequence was determined by direct sequencing of the PCR product and was performed by CeMIA SA, Larissa, Greece. Analysis of the sequence was performed with the basic sequence alignment BLAST program (National Center for Biotechnology Information, U.S. National Library of Medicine, Bethesda MD, USA) run against the database provided on the website of the National Center for Biotechnology Information (http://www.ncbi.nlm.nih.gov/BLAST, National Center for Biotechnology Information, U.S. National Library of Medicine, Bethesda MD, USA). Phylogenetic trees based on the nucleotide sequences of the 16S rRNA gene fragments were constructed with the Molecular Evolutionary Genetics Analysis software version 7.0 (Pennsylvania State University, State College, PA, USA) [36] using the neighbor-joining algorithm (1000 bootstrap replication).

### 2.7. Nucleotide Sequence Accession Numbers

The nucleotide sequence data have been submitted to the European Nucleotide Archive GenBank database under accession numbers LR027392 to LR027460 (16S rRNA sequences), BioProject: PRJEB28499.

### 2.8. Statistical Analysis

The comparisons between the corresponding FBS^0^ crop and F crop values in each case were performed using one-way ANOVA and Tukey’s honest significant difference post hoc test at *p* < 0.05.

## 3. Results

### 3.1. Dry Mass Accumulation per Plant

**Figure 2:**
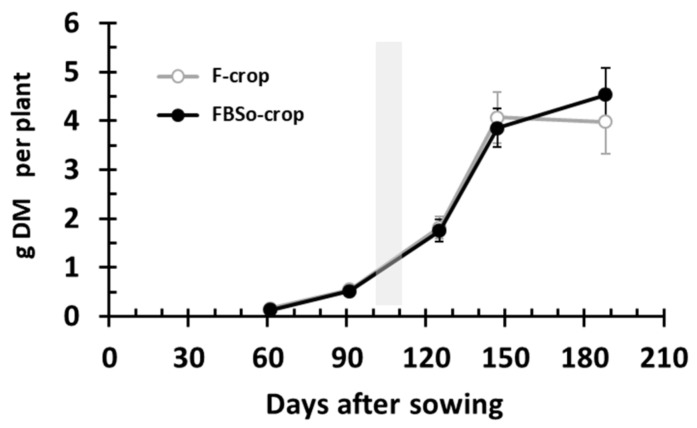
Dry mass accumulation per plant. The gray histogram indicates the topdressing application. Bars indicate standard deviation. An asterisk indicates a statistically significant difference (*p* < 0.05).

Despite the fact that the FBS^0^ crop was grown in soil containing 53% less phosphorus (Table 1), the dry mass accumulation per plant was statistically the same as the F crop’s one (Figure 2). Moreover, a tendency for higher accumulation (though not statistically significant) was observed at 189 DAS. The FBS^0^ crop had a 3.4% lower yield (4.89 t ha^−1^) compared to the F crop (5.06 t ha^−1^).

### 3.2. Developmental Dynamics of Organic Nitrogen versus Organic Sulfur in the Crop’s Aerial Part

**Figure 3:**
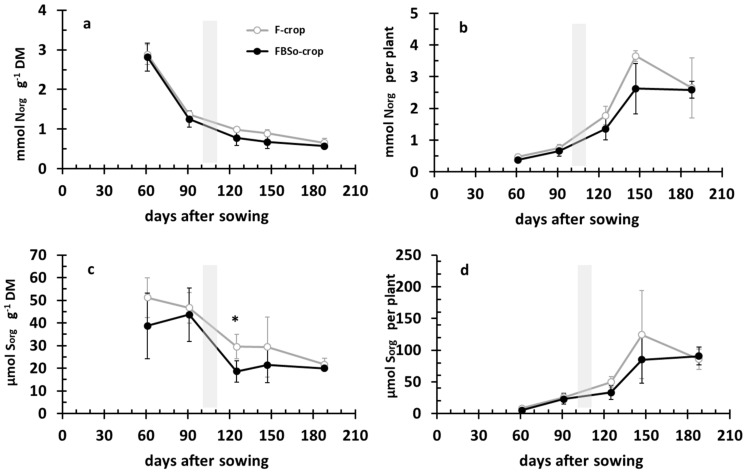
Time course of organic nitrogen (N_org_; (**a**)) and organic sulfur (S_org_; (**c**)) concentration along with the corresponding accumulations per plant in the crop’s aerial part (**b**,**d**). The gray histogram indicates the application of topdressing. Bars indicate standard deviation. An asterisk indicates a statistically significant difference (*p* < 0.05).

Dynamics of both N_org_ and S_org_ concentrations followed the same pattern in both crops. Patterns of both nutrients in the FBS^0^ crop had a tendency to be lower, but the difference was not statistically significant through development. As a result, both N_org_ and S_org_ accumulations per plant in the aerial part followed a sigmoid curve, with a tendency to be lower in the FBS^0^ crop, especially after topdressing, although this difference was not statistically significant (Figure 3).

### 3.3. Quantification of Cultivable ARS-Producing Bacterial Communities in the Rhizosphere of F- and FBS^0^-Treated Wheat

**Figure 4:**
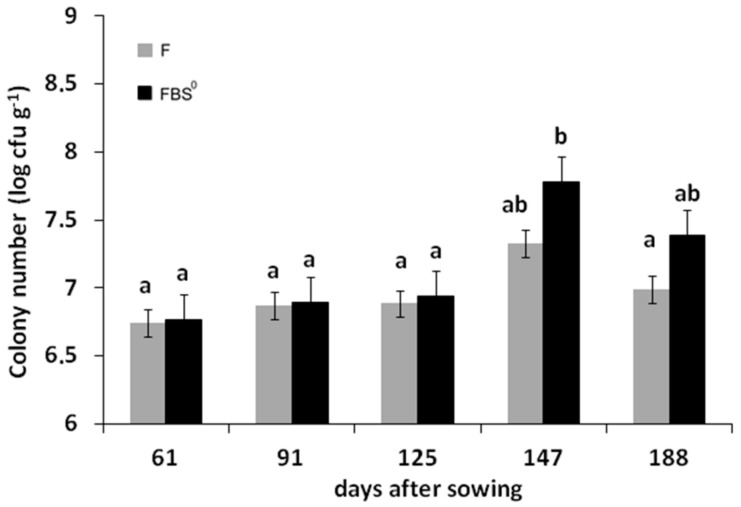
Abundance (cfu per g^−1^ dry mass) of rhizospheric soil aerobic bacteria growing in M9-Xsulf minimal media at 61, 91, 125, 147, and 188 DAS for both (F and FBS^0^) fertilization regimes.

**Figure 5:**
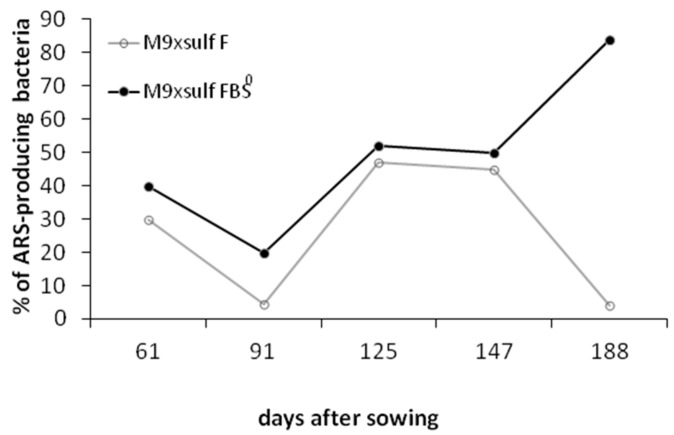
Abundance (percentage of ARS-producing bacteria in the total population of aerobic cultured bacteria growing in M9-Xsulf minimal media) in the F and FBS^0^ crops during development.

Rhizospheric soil (Rhs) samples collected from the FBS^0^ and F crops from 61 to 188 DAS were evaluated for total cultured bacterial population. Estimations of CFU per gram of dry Rhs were carried out in full media (nutrient broth agar) and minimal media (M9 mineral medium agar). The average total population of bacterial heterotrophs was similar at 61, 91, and 125 DAS and reached a higher density at 147 DAS in both the FBS^0^ and F crops (Figure 4). However, the heterotrophic bacterial population at 147 DAS was notably higher in FBS^0^ as compared to the F crop (Figure 4), suggesting that S^0^ may have a positive effect on the population density of rhizospheric bacteria. Examination of the ARS-producing bacterial communities (blue colonies in M9 X-sulf medium) in all Rhs samples revealed that the relative abundance of ARS-possessing bacteria was radically higher in FBS^0^- than in F-treated wheat. The densities of ARS-producing bacteria changed during the course of development of both FBS^0^- and F-treated wheat (Figure 5). In the FBS^0^ crop at 61 DAS, the ARS-possessing bacteria community represented 40% of the heterotrophs. At 125 and 147 DAS, almost 50% of the heterotrophs were identified as ARS-possessing bacteria, while at 188 DAS the ARS-possessing bacteria constituted the majority (84%) of the heterotrophs. In the F crop at 61 DAS, the ARS-producing bacteria represented 30% of the heterotrophs; this percentage was reduced to 4% at 188 DAS. Interestingly, at 61 DAS in the FBS^0^ crop, the ARS-possessing bacteria represented about 40% and reached at 188 DAS 85% of the heterotrophs (Figure 5). Taken together, these results suggest that mixing conventional fertilizers with S^0^ induces a noteworthy shift in the population density of ARS-possessing bacteria (i.e., the percentage of ARS-possessing bacteria in the total bacterial population), thus enhancing the levels of bioavailable sulfur supply to the plant.

### 3.4. Diversity of ARS-Producing Bacteria

**Figure 6:**
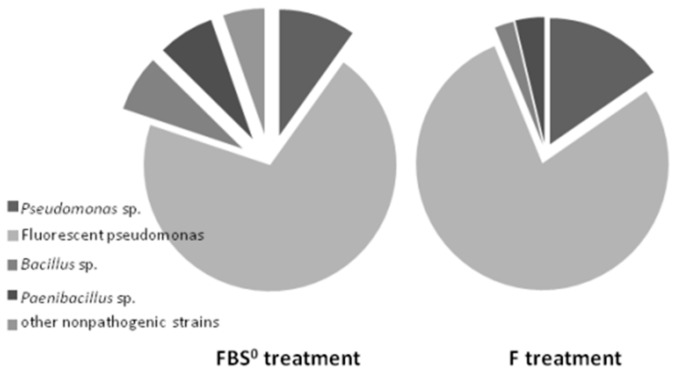
Relative abundance of ARS-producing bacterial isolates’ phylotypes at the genus level in the FBS^0^ versus F crop’s rhizosoil. The phylogenetic position of the bacterial isolates was based on 16S rRNA sequences.

**Figure 7:**
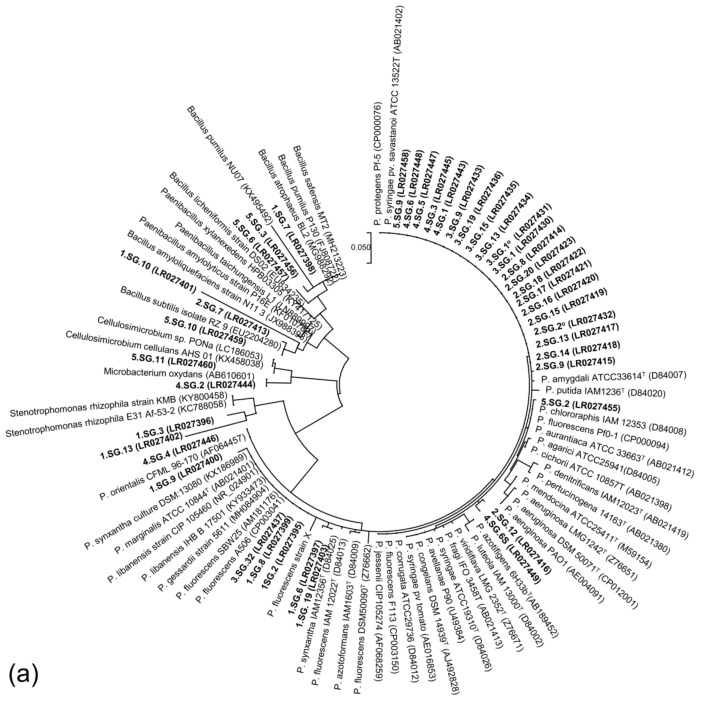

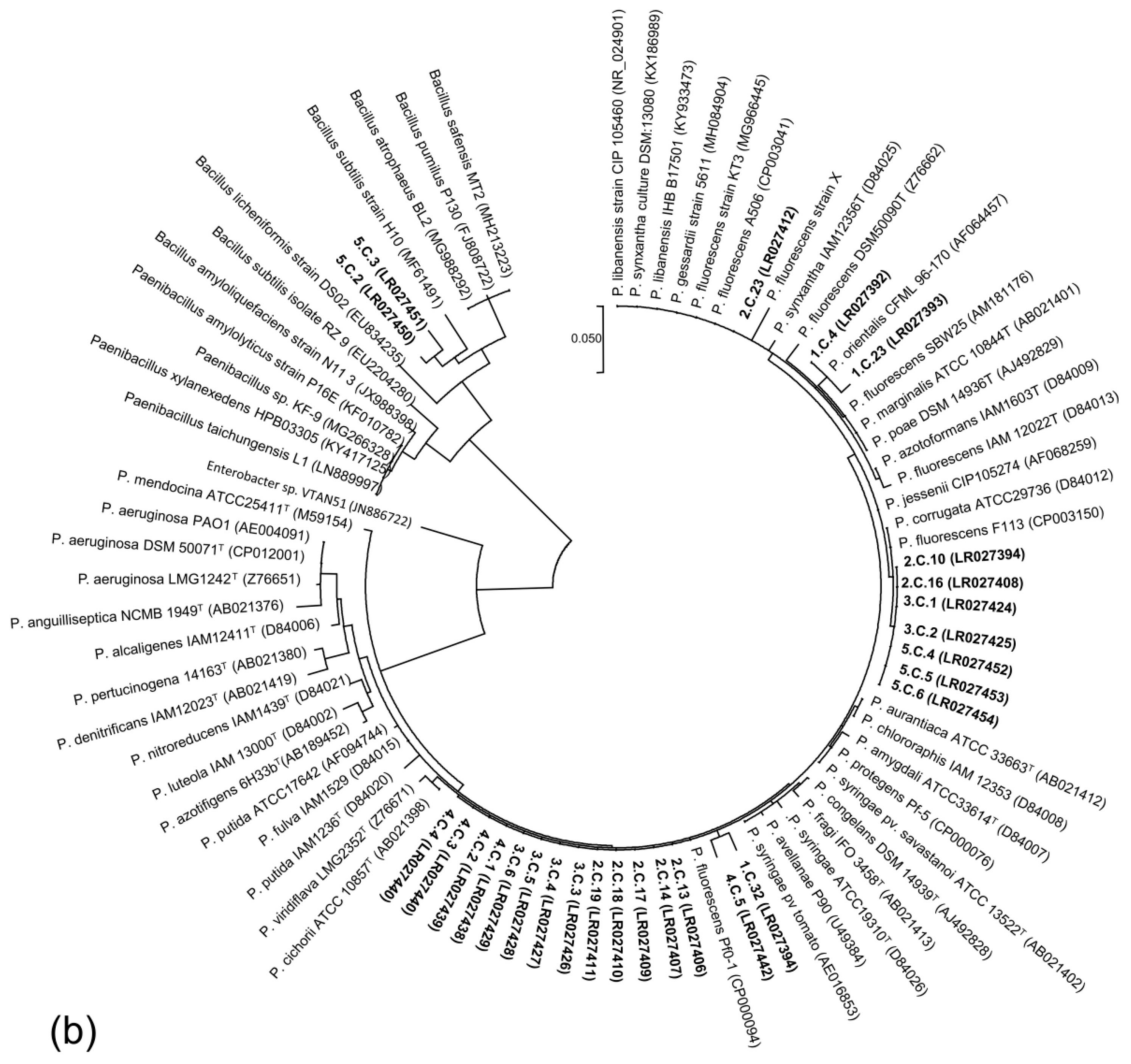
Phylogenetic position of ARS-producing bacterial isolates based on their 16S rRNA sequences. (**a**) FBS^0^ treatment and (**b**) F treatment. List of ARS-producing bacterial isolates and their characteristics are presented in Table S1.

The overall bacterial community was further characterized by sequencing the 16S rRNA gene of the 68 ARS-producing bacterial strains recovered from the examined phenological growth stages of wheat: 27 strains from the F crop and 41 strains from the FBS^0^ crop. The ARS-producing bacterial isolates per sampling day and their characteristics from both the F and FBS^0^ treatment are provided in Table S1. A data analysis revealed that the ARS-producing bacterial community associated with Rhs in both the F and FBS^0^ crops was dominated by bacteria of the *Pseudomonas* genus, by 90% and 77%, respectively (Figure 6). Almost 80% of the identified ARS-producing bacterial isolates that were classified as *Pseudomonas* sp., *Pseudomonas koreensis*, or *P. fluorescens* showed fluorescent pigment production when they were tested on King medium B. The second most dominant group belonged to the *Bacillus* genus, whereas bacteria belonging to the *Cellulomonas*, *Acinetobacter*, *Stenotrophomonas*, and *Xanthomonas* genera were also found among the F- and FBS^0^-treated wheat isolates (Figure 6). When two neighbor-joining trees were created, based on the 16S rRNA sequences from ARS-producing bacterial isolates (one for each treatment) (Figure 7), it was noticed that the identified ARS-producing bacterial isolates were classified among the clades of beneficial *Pseudomonas*, *Bacillus*, and other nonphytopathogenic antagonistic strains deposited in public databases.

### 3.5. Phosphorus Dynamics in the Rhizosoil and the Aerial Part of the Plant 

**Figure 8:**
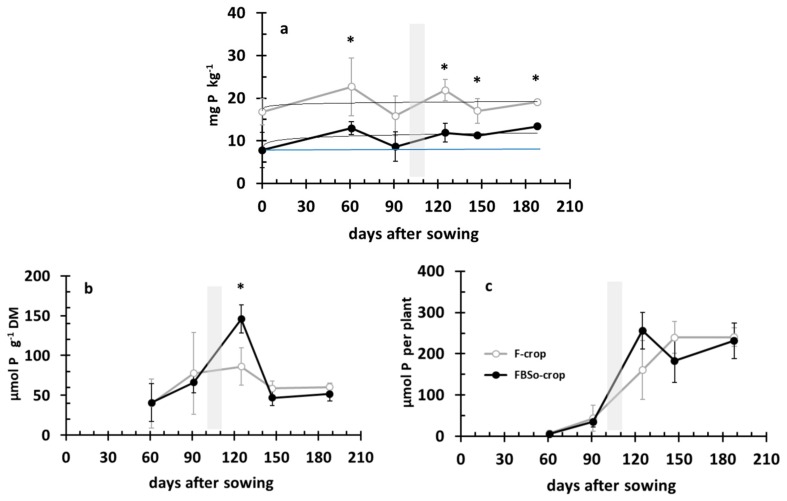
Phosphorus dynamics (**a**) in the rhizosoil (Olsen P concentration) and in the aerial part: (**b**) P concentration; (**c**) P accumulation, during crop development. Bars indicate standard deviation. An asterisk indicates a statistically significant difference (*p* < 0.05).

During development, the Olsen P concentration of rhizosoil fluctuated around 10 mg kg^−1^ in the FBS^0^ crop and 18 mg kg^−1^ in the F crop (Figure 8a). It is noticeable that the fluctuation pattern was the same in both treatments. In the aerial part of FBS^0^ plants, the P concentration was 75% higher than in F plants at 125 DAS, i.e., after topdressing. Then, there was a tendency for a lower P concentration; however, it was not statistically significant (Figure 8b). Following this concentration pattern, P accumulation per plant presented the same sigmoid pattern as the F treatment. P concentration in the F crop increased up to 125 DAS and then decreased and stabilized to 60 μmol gDM^−1^. The same pattern was followed by the FBS^0^ treatment. P accumulation in the FBS^0^ crop tended to be higher at 125 DAS; however, this difference was not statistically significant. Both curves reached a plateau at the same level (Figure 8c).

### 3.6. Iron Dynamics in the Rhizosoil and the Aerial Part of the Plant 

**Figure 9:**
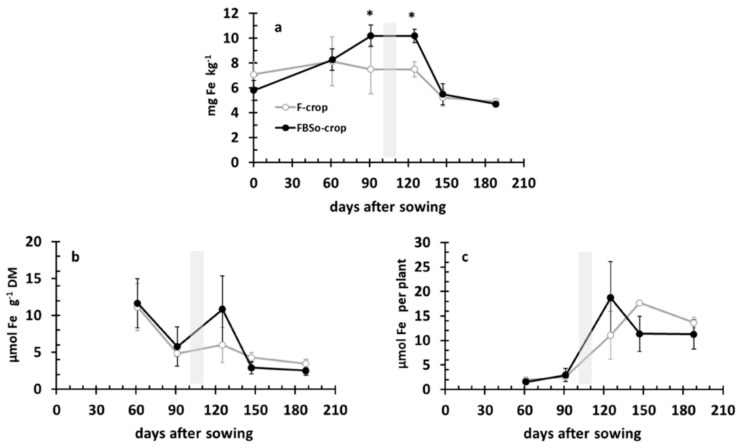
Iron dynamics in the rhizosoil (Fe-DTPA concentration (**a**) and in the aerial part (Fe concentration, (**b**); Fe accumulation (**c**) during crop development. Bars indicate standard deviation. An asterisk indicates a statistically significant difference (*p* < 0.05).

Crops started with 7.08 mg kg^−1^ of Fe in the F crop’s soil and 5.78 mg kg^−1^ of Fe (i.e., 18.4% less) in the FBS^0^ crop’s one (Table 1). During development, Fe remained almost stable in the F crop’s RS, up to 125 DAS, and then decreased to 5 mg kg^−1^. Instead, the Fe concentration in the FBS^0^ crop’s RS increased up to 10 mg kg^−1^ before topdressing and stabilized at 125 DAS. Then, a significant decrease to the F crop’s levels took place (Figure 9a). In the aerial part of the plant, the Fe concentration decreased from 12 μmol gDM^−1^ to 5 between 61 and 91 DAS, when it more or less stabilized (Figure 9b). FBS^0^ treatment presented a differentiated pattern. After topdressing, the Fe concentration increased to the levels of 61 DAS and then decreased and stabilized at 3 mg kg^−1^ (Figure 9b). Fe accumulation followed a sigmoid pattern in the F crop, while the FBS^0^ crop presented a differentiated one. More specifically, after topdressing (125 DAS), Fe accumulation tended to be higher and then dropped and tended to remain lower towards the end of the crop cycle (Figure 9c).

### 3.7. Correlations between S_org_, Fe, P, and N_org_ Crops’ Nutritional Dynamics

**Figure 10:**
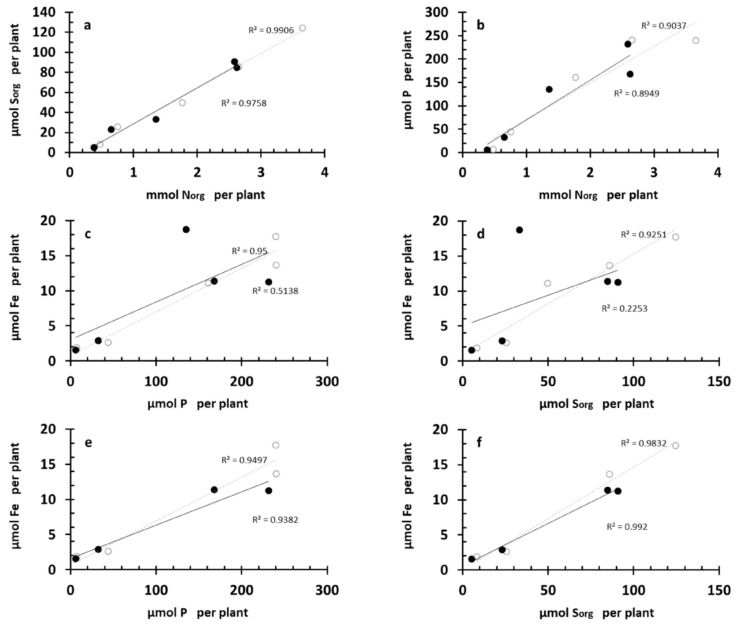
Correlations between the corresponding values of N_org_ and S_org_ (**a**), N_org_ and P (**b**), P and Fe (**c**,**e**), S_org_ and Fe (**d**,**f**) accumulations per plant. (**e**,**f**) were produced by eliminating the point representing 125 DAS (explanation in the text).

N_org_ and S_org_ accumulations per plant in the F crop had a highly linear relationship (*R*^2^ = 0.9906). The FBS^0^ treatment presented a strong linear relationship, too (*R*^2^ = 0.9758), with the same slope (Figure 10a), suggesting that the treatment did not disturb the relationship between N_org_ and S_org_ throughout crop development.

The correlation between N_org_ and P accumulation per plant (Figure 10b) was strongly linear (*R*^2^ = 0.9037) in the F crop. This held true in the FBS^0^ crop (*R*^2^ = 0.8949), too, with a tendency for a higher slope, suggesting enhanced accumulation of P in relation to N_org_ after topdressing.

Comparing the accumulation of Fe with that of P or S_org_ per plant in the F crop, strong linear relationships were obtained, with *R*^2^ = 0.95 and 0.9251, respectively. The treatment disturbed these linear correlations (Figure 10c,d). By eliminating the point representing 125 DAS, the strong linear relationship was restored (*R*^2^ = 0.9382 and 0.992, respectively; Figure 10e,f).

### 3.8. Phosphate Solubilization, and/or Siderophore-Producing Capacity, and/or Ureolytic Activity of the ARS-Producing Bacterial Isolates

**Figure 11:**
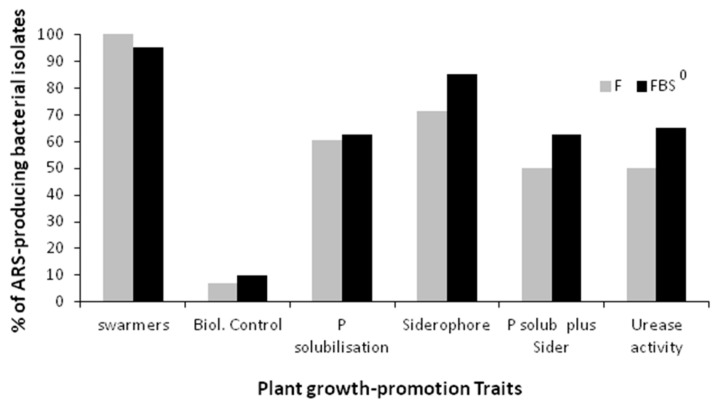
Percentage of 68 ARS-producing bacterial isolates’ plant growth-promotion traits. The 68 bacterial isolates from F- and FBS^0^-treated wheat rhizosphere were further tested for plant growth-promoting traits (swarming motility, biological control, phosphate solubilization, siderophore production, urea hydrolysis) in vitro. P solub + Sider represents the percentage of bacterial isolates possessing both siderophore production and phosphate solubilization activity.

**Figure 12:**
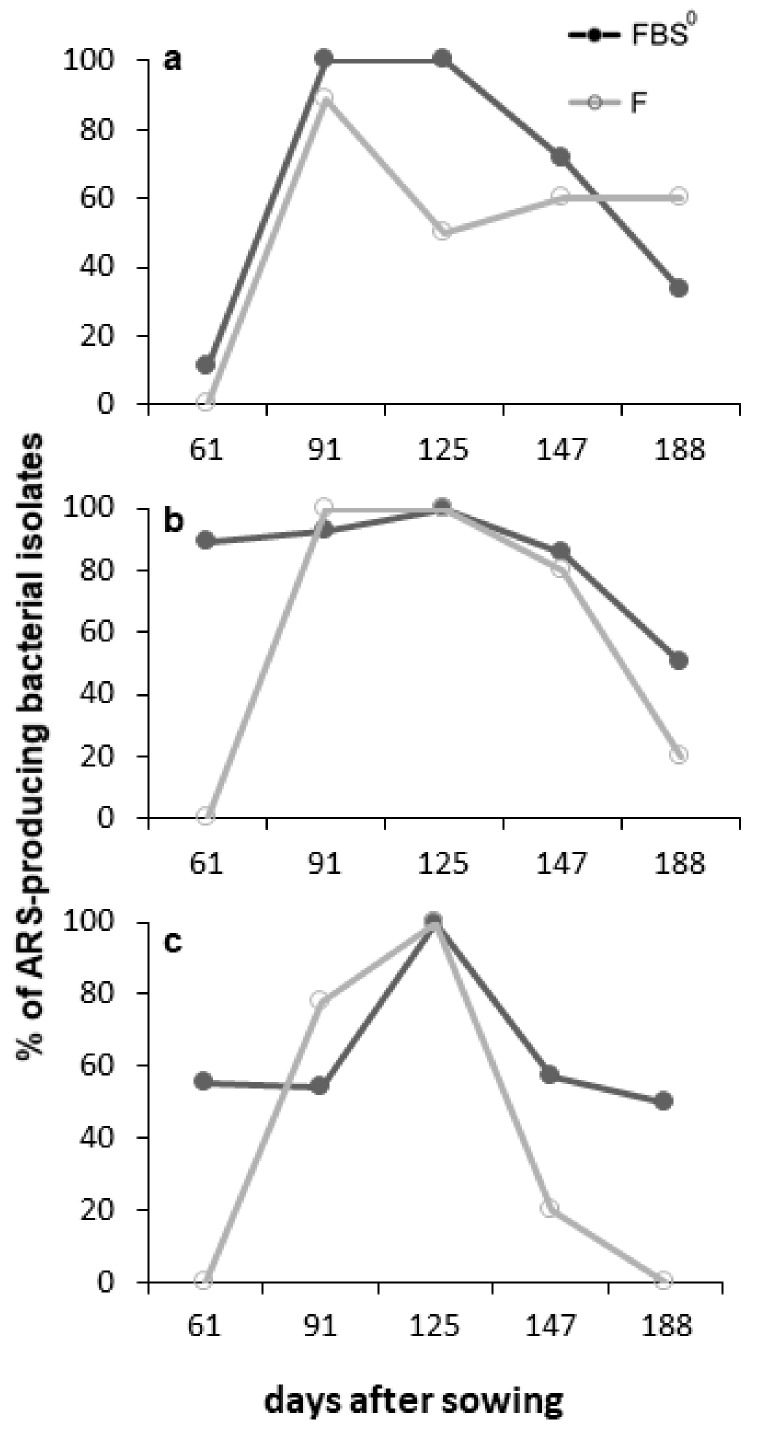
Proportions of 68 ARS-producing bacterial isolates possessing (**a**) P solubilization activity, (**b**) siderophore production, and (**c**) urease production in the total population of F- and FBS^0^-treated wheat at each developmental stage.

The ARS-producing bacterial isolates collected from both the F and FBS^0^ crops in each developmental stage were further examined to see whether they possess additional PGP traits related to P solubilization, siderophore production, swarming motility, urease production, and antagonistic activity against phytopathogenic fungi (Figure 11). Interestingly, almost all isolates showed a swarming ability, whereas a significant proportion of the isolates collected from both the F and FBS^0^ crops, 50% and 62% respectively, possessed both traits and involved P and Fe mobilization (Figure 11). The incorporation of low levels of S^0^ had a noteworthy effect on the relative abundance of ureolytic bacterial isolates; a much larger proportion (66%) of isolates collected from the FBS^0^ treatment exhibited ureolytic activity as compared to the F treatment (50%) (Figure 11). The proportion of ARS-producing bacteria possessing P or Fe mobilization or ureolytic activity changed during the development of both the F and FBS^0^ crops (Figure 12). Interestingly, at 61 DAS, a small fraction (10%) of isolates collected from the FBS^0^ crop possessed P-solubilizing capacity, whereas at 91 DAS this proportion rose to over 90%, while at 61 and 91 DAS the vast majority (90–100%) of isolates were found to produce siderophores (Figure 12a,b). In F-treated wheat plants at 61 DAS, a negligible fraction of ARS-producing bacteria possessed P or Fe mobilization activity, while at 91 DAS over 90% of the isolates possessed both traits. Taken together, these results suggest that at 61 and 188 DAS the growing wheat plants were selectively recruiting ARS-producing bacteria possessing both traits, and thus may enhance the levels of bioavailable phosphate and iron to the crop. At subsequent developmental stages of the plant, the abundance of ARS-producing bacteria possessing one or both traits were gradually decreasing, but not eliminated, suggesting that this group of rhizospheric bacteria may continuously provide bioavailable P and Fe to the crop plants, although at gradually reduced rates.

The distribution of ureolytic bacteria also changed during the development of both the F and FBS^0^ crops; at 61 DAS, none of the isolates collected from F-treated wheat showed ureolytic activity, whereas a large fraction (60%) of isolates collected from FBS^0^ wheat were positive for ureolytic activity. After topdressing, at 125 DAS, all the isolates collected from both the F and FBS^0^ crops showed positive ureolytic activity, but at subsequent developmental stages of the plant this gradually decreased (Figure 12c).

## 4. Discussion

The aforementioned data supported the working hypothesis: The FBS^0^ crop provided a 3.4% lower yield compared to the F crop; therefore, the FBS^0^ treatment significantly alleviated the potential yield loss due to the low amount of available phosphorus in the soil, clearly showing that the incorporation of 2% elemental sulfur in the conventional fertilizer granules helped the crop to effectively counteract the inferior quality of the rhizosoil. Moreover, the interactions between P, Fe, S_org_, and N_org_ were sustained, while the rhizospheric microbiome played a remarkable role in sustaining them. The interactions between iron nutrition and sulfur nutrition have received increasing attention in various plant species, with various approaches and at various levels (e.g., [37,38,39,40,41]). Significant interactions between plant Fe acquisition mechanisms and external sulfate supply have been reported [42]. Lower leaf concentrations have been observed in sulfur-deficient plants in relation to sufficient sulfur treatment [43]. The low availability of sulfate could affect the accumulation and release of phytosiderophores in Fe-deficient barley roots, associated with a possible impairment of Fe acquisition in these plants. These results suggest that the requirement of S may be higher when plants are experiencing Fe deficiency, and that plant responses to Fe deficiency are modified by S supply [42].

The nature of the FBS^0^ fertilizer has been studied [27]. Briefly, the FBS^0^ fertilizer used in this experiment contained sulfate as the accompanying anion of ammonium (i.e., directly available S), along with 2% S^0^ (not directly available S). The ingredients of the binder (i.e., a mixture of molasses and glycerol) are both water-soluble and can be used by soil microorganisms, along with S^0^, thus suggesting microbial action around the granules [26,27]. In this paper we show that the aforementioned interactions are highly affected by the rhizospheric microbiome under the circumstances.

### 4.1. The Addition of Elemental Sulfur to the Fertilization Scheme Promoted a Higher Density of ARS-Producing Bacterial Population in the Rhizosphere

In the present study we demonstrated that the rhizosphere of FBS^0^-treated wheat harbors a higher density of ARS-producing bacterial population, at all stages of plant development, as compared to conventional F-treated wheat. Therefore, we focused on the ARS-producing bacterial population in the rhizosphere and its phosphate and/or iron solubilization capacity, along with ureolytic activity, towards understanding the nutritional dynamics of the rhizosphere and aerial part of the plants with regard to organic sulfur, iron, phosphorus, and organic nitrogen. It has been established that PGPR, associated with the rhizosphere of cultivated plants, influence nutrient bioavailability in soil and nutrient uptake by the plant [44]. The abundance of ARS-producing bacteria appears to be primarily influenced by the incorporation of low levels of S^0^ into conventional fertilizer, as well as the crop’s developmental stage. The growth of ARS-producing bacteria is promoted by the presence of S in organic form in the soil and its activity may not be repressed by inorganic sulfate [25]. Soil amending with plant residues, wheat, fescue, and mustard, exhibiting different C:S ratios, revealed that residues with high S content significantly stimulated the population density of ARS-producing bacteria as compared to those with low S content [45]. Similar findings were also reported in [46], demonstrating that rhizosphere soil arylsulfatase activity was influenced by the fertilization regime; the application of large amounts of manure and mineral fertilizer resulted in higher soil arylsulfatase activity than mineral fertilizers or the unfertilized control. The fact that S application to the soil has a positive effect when coupled with organic fertilization is pivotal for low-input or organic systems. This has been shown for durum wheat in a Mediterranean environment [47], where it has been showed that S application significantly increased grain yield under organic rather than mineral fertilization, and the present study provides a justification for this.

Furthermore, it has been demonstrated that enhancement of the population density of rhizobacteria that are able to transform organic sulfur into bioavailable forms promoted plant fortification with S of *Lolium perenne* cultivated in acid soil [23]. The involvement of ARS-producing bacteria in S mobilization is further corroborated by two independent studies using plants cultivated in calcareous soils: Grecut et al. (2009) [22] demonstrated that S-demanding plants, such as rape, appear to recruit higher numbers of ARS-producing bacteria as compared to barley. Vong et al. (2007) [48] demonstrated that rape took up 2–3 times more S derived from fertilizer than barley, resulting in a significant reduction in soil S bioavailable pools at 63 DAS. Thus, ARS-producing bacteria represent a new class of potential biofertilizers that may have enhanced organic S mobilization and provide a sustained input of sulfate, irrespective of the sulfate added by the fertilizer, during crop development. In calcareous soils, S from added ammonium sulfate fertilizer probably reacts with CaCO_3_ to precipitate CaSO_4_ [49,50], and since CaSO_4_ is slightly soluble in water [50], sulfate derived from fertilizers added to calcareous soils may become partly available for plant uptake, depending on the irrigation and precipitation. Thus, the action of ARS-producing bacteria may be important, in particular in unirrigated field crops, for maintaining a sustained input of bioavailable S to the plant.

### 4.2. A Large Fraction of the ARS-Producing Bacterial Isolates Possessed Phosphate Solubilization Capacity

The aforementioned data also demonstrated that a large fraction of the ARS-producing bacteria possessed P-mobilizing capacity, which may have enhanced P mobilization, leading to plant growth promotion. Most of the accumulated P applied to the soil remains in nonbioavailable forms, with P mobilization (measured as Olsen *p* values) ranging from 9 to 49 mg Kg^−1^ [51], thus making the enhancement of bioavailable P a formidable task. Recent studies have shown that artificial enhancement of soil population density of calcareous soils with P-solubilizing bacteria (PSB) enhances the release of Olsen *p* values of natural calcareous soils or calcareous soils amended with mineral or organic fertilizers [10]. On the other hand, sewage sludge is an alternative P source to P fertilizer derived from rock phosphate [52]. Our data demonstrated that, regardless of the differences in Olsen *p* values of the FBS^0^- and F-treated wheat RS, the P concentration in the FBS^0^ crop at 125 DAS was significantly higher as compared to the F crop, suggesting a more efficient uptake of P by the FBS^0^ crop after topdressing. The higher P concentration in the FBS^0^-treated wheat may be a consequence of the high population density of PSB in the FBS^0^ crop’s Rhs; the high density of PSB at 91 and 125 DAS enhanced the P mobilization and provided a sustained input of bioavailable phosphate for uptake by the plant. These findings are in close agreement with recent studies wherein it has been demonstrated that the inoculation of wheat grown in different types of alkaline soils (with Olsen *p* values ranging from 4.8 to 8.7 mg kg^1^) with P-solubilizing *B. subtilis* increased P uptake the more the Olsen *p* values in the soil decreased [13]. Furthermore, several studies have shown that inoculation with PSB increased growth and P nutrition in strawberries [53], broad beans [54], and *Zea mays* [55] grown in calcareous soil, rendering the exploitation of PSB a formidable task in improving agricultural productivity.

### 4.3. A Large Fraction of the ARS-Producing Bacterial Isolates Possessed Siderophore-Producing Capacity

The data from this study also demonstrated that a large fraction of the ARS-producing bacteria possessed Fe-mobilizing capacity, which may have enhanced Fe mobilization, leading to plant growth promotion. The Fe concentration of the FBS^0^ crop’s RS increased up to 125 DAS, while the F crop’s one gradually decreased. However, the FBS^0^ crop’s rhizosphere was more abundantly populated by ARS-producing bacteria possessing siderophore-producing capacity as compared to the F-treated crop. The high population density of siderophore-producing bacteria may counteract the soil calcification, thus generating more bioavailable Fe in the FSB^0^ as compared to the F crop’s rhizosphere, which can explain the enhanced concentration of iron in FBS^0^-crop at 125 DAS as compared to the F crop. The FBS^0^ and F crops’ RS Fe concentration was related to the population density of siderophore-producing bacteria in the Rhs during the crop’s cycle; a drop in the population density of siderophore-producing bacteria at 149 and 191 DAS coincided with a decrease in the RS Fe concentration and a significant drop in the aerial part’s Fe concentration, which was reflected in the overall Fe accumulation in wheat. This may point to the idea of artificial enhancement of a crop’s RS population density at the proper time, as the addition of a mixture of selected ARS-producing bacterial isolates possessing siderophore-producing capacity to the RS can affect the final Fe uptake and accumulation. Artificial enhancement of the wheat Rhs population density by inoculation with siderophore-producing bacteria significantly increased the Fe content of wheat and rice [6,56]. Similarly, the inoculation of fertilized wheat with PGPR improved the growth, yield, and nutrient uptake [57]. The inoculation of peanuts cultivated in calcareous soils with bacteria producing siderophores alleviated the antagonistic effect of calcareous soils and increased the bioavailable content of Fe in soil, thus promoting iron nutrition [15].

### 4.4. A Large Fraction of the ARS-Producing Bacterial Isolates Possessed Ureolytic Activity

Furthermore, our data demonstrated that the abundance of ARS-producing bacterial population possessing ureolytic activity was significantly affected by fertilization treatments; the FBS^0^ fertilization scheme resulted in a higher population density of ureolytic bacteria as compared to the F one. The fate of urea mainly depends on bacterial urease activity, which is also considered an indicator of soil quality [58] d. As urease converts urea to ammonium, which is either taken up by the plant roots or further processed to nitrate by soil microorganisms, the high population of ureolytic bacteria in FBS^0^-treated wheat may positively affect the N nutritional status of plant. The incorporation of the urease inhibitor NBPT along with 2% elemental sulfur as ingredients in the fertilization schemes resulted in even higher yields in similar experiments [27]. This supports the hypothesis that NBPT suppressed the ureolytic activity of the ARS-producing bacterial isolates, allowing for better ammonium utilization.

### 4.5. Towards a Sustainable Agronomic Biofortification Practice

In addition to S assimilation and its effects on the yield and quality of wheat [59], the effect of S rate on durum wheat grain yield and quality has been studied under Mediterranean conditions [60]. In this experiment, a very small amount of S^0^ (5.5 kg ha^−1^) was added as an ingredient during fertilization at sowing and the practice had a positive impact on the rhizospheric bacteria of the calcareous soil. Obviously, this is the case with related experiments, too [26,27]. By extension, this fostered the idea that small amounts of S^0^ fine powder could be applied alone in low-input or organic agricultural systems in order to trigger the same result. On the other hand, given that the FBS^0^ crop is biofortified as regards the studied nutrients, these data demonstrate the merit of deploying inocula composed of the isolated strains from this experiment, or mixtures of these strains as a biofortification strategy for wheat, particularly under calcareous soil conditions. The compatibility issue with multi- versus single-strain plant growth promoting microbial inoculants has been discussed [61]. Taking into account the interactions between carbon, nitrogen, phosphorus, sulfur, and iron biogeochemical cycles, as discussed in [62], the application of such rates of S^0^ by itself, or coupled with proper single- or multistrain inocula of ARS-producing bacteria, might effectively sustain these interactions, thus suggesting a sustainable agronomic biofortification approach that merits further investigation.

## 5. Conclusions

The incorporation of low levels (2% w/w) of S^0^ as ingredient of fertilizer granuls in the fertilization scheme of durum wheat grown on a calcareous soil containing minimal available P increased the population density of the rhizospheric ARS-producing bacteria, a large fraction of which also possessed P- and Fe-mobilizing capacity, along with ureolytic activity. The changes induced in the rhizospheric microbiome promoted plant growth, fortified the crop’s P, Fe, organic S, and organic N contents, and increased the grain yield.

## Figures and Tables

**Table 1:** Soil quality of the experimental fields at sowing.

Crop	Clay	Silt	Sand	Class	pH	EC	CaCO_3_	SOM
	%	%	%			μS cm^−1^	%	%
F	38	34	28	Silty Clay	7.86	685	31.5	2.55
FBS^0^	42	30	28	Clay	8.01	613	24.5	1.79
Δx/x (%)					1.9	−10.5	−22.2	−29.8
	**Olsen P**	**NO_3_-N**	**Kexc**	**Fe-DTPA**	**Mn-DTPA**	**Cu-DTPA**	**Zn-DTPA**	
	mg kg^−1^	mg kg^−1^	mg kg^−1^	mg kg^−1^	mg kg^−1^	mg kg^−1^	mg kg^−1^	
F	16.8	28.48	410	7.08	8.97	1.76	1.26	
FBS^0^	7.8	24.57	270	5.78	6.25	1.43	0.75	
Δx/x (%)	−53.6	−13.7	−34.1	−18.4	−30.3	−18.8	−40.5	

**F crop**: the crop that was subject to conventional fertilization (F) treatment according to the local agricultural practices; **FBS^0^ crop**: the crop that received the corresponding FBS^0^ treatment; **EC**: electric conductivity; **Kexch**: soil exchangeable potassium. **SOM:** soil organic matter; **DTPA**: diethylenetriaminepentaacetic acid.

## Supplementary Materials

The following are available online at https://www.mdpi.com/2223-7747/8/10/379/s1. Table S1, List of ARS-producing bacterial isolates and their characteristics, isolated from F treatment (control) and FBS^0^ treatment.
