# Supplementary material for: Impact of Elemental Sulfur on the Rhizospheric Bacteria of Durum Wheat Crop Cultivated on a Calcareous Soil
Source: Plants (Basel). 2019 Sep 27;8(10):379. doi: 10.3390/plants8100379 (PMC6843240; doi:10.3390/plants8100379)
Supplement: Supplementary file 1 [file plants-08-00379-s001.pdf]

Table S1. List of ARS-producing bacterial isolates and their characteristics, isolated from F- and FBS^0-crop's rhizosphere.

		DAS	Isolate	P	Fe	U	Fluo	Biocontrol	16S rRNA seq	Accession Number
F- crop	1	**61**	**1.C.4**	-	-	-	-	-	*P. orientalis*	LR027392
	2		**1.C.23**	-	-	-	+	-	*P. orientalis*	LR027393
	3		**1.C.32**	-	-	-	+	-	*P. fluorescens*	LR027394
	4	**91**	**2.C.10**	+	+	-	+	-	*P. thivervalensis*	LR027405
	5		**2.C.13**	+	+	+	+	-	*P. moraviensis*	LR027406
	6		**2.C.14**	+	+	+	+	-	*P. moraviensis*	LR027407
	7		**2.C.16**	+	+	+	+	-	*P. thivervalensis*	LR027408
	8		**2.C.17**	+	+	+	+	-	*P. koreensis*	LR027409
	9		**2.C.18**	-	+	+	+	-	*P. koreensis*	LR027410
	10		**2.C.19**	+	+	+	+	+	*P. moraviensis*	LR027411
	11		**2.C.23**	+	+	+	+	+	*P. fluorescens*	LR027412
	12	**125**	**3.C.1**	-	+	+	-	-	*P. thivervalensis*	LR027424
	13		**3.C.2**	-	+	+	-	-	*P. fluorescens*	LR027425
	14		**3.C.3**	-	+	+	+	-	*P. koreensis*	LR027426
	15		**3.C.4**	+	+	+	+	-	*P. fluorescens*	LR027427
	16		**3.C.5**	+	+	+	+	-	*P. koreensis*	LR027428
	17		**3.C.6**	+	+	+	+	-	*P. koreensis*	LR027429
	18	**147**	**4.C.1**	+	+	-	+	-	*P. moraviensis*	LR027438
	19		**4.C.2**	-	+	-	+	-	*P. koreensis*	LR027439
	20		**4.C.3**	-	-	-	+	-	*P. koreensis*	LR027440
	21		**4.C.4**	+	+	+	+	-	*P. koreensis*	LR027441
	22		**4.C.5**	+	+	-	+	-	*P. fluorescens*	LR027442
	23	**188**	**5.C.2**	+	+	-	-	-	*Bacillus sp*	LR027450
	24		**5.C.3**	-	-	-	-	-	*P. megaterium*	LR027451
	25		**5.C.4**	+	-	-	-	-	*P. thivervalensis*	LR027452
	26		**5.C.5**	-	-	-	+	-	*P. thivervalensis*	LR027453
	27		**5.C.6**	+	-	-	-	-	*P. thivervalensis*	LR027454

		DAS	Isolate	P	Fe	U	Fluo	Biocontrol	16S rRNA seq	Accession Number
**FBS^0-crop**	1	**61**	**1.SG.2**	-	+	-	+	-	*P. fluorescens*	LR027395
	2		**1.SG.3**	-	+	-	-	-	*Stenotrophomonas rhizophila*	LR027396
	3		**1.SG.6**	-	+	+	+	-	*P. fluorescens*	LR027397
	4		**1.SG.7**	-	+	+		-	*Bacillus sp.*	LR027398
	5		**1.SG.8**	-	+	+	+	-	*P. fluorescens*	LR027399
	6		**1.SG.9**	+	+	+	+	-	*P. azotoformans*	LR027400
	7		**1.SG.10**	-	+	+	-	-	*Paenibacillus amylolyticus*	LR027401
	8		**1.SG.13**	-	+	-	+	-	*Stenotrophomonas rhizophila*	LR027402
	9		**1.SG.19**	-	-	-	+	-	*P. fluorescens*	LR027403
	10	**91**	**2.SG.7**	+	+	+	+	-	*P. koreensis*	LR027413
	11		**2.SG.8**	+	+	+	+	-	*P. koreensis*	LR027414
	12		**2.SG.9**	+	+	+	+	-	*P. koreensis*	LR027415
	13		**2.SG.12**	+	+	-	+	-	*P. koreensis*	LR027416
	14		**2.SG.13**	+	+	-	+	-	*P. koreensis*	LR027417
	15		**2.SG.14**	+	-	-	+	-	*P. koreensis*	LR027418
	16		**2.SG.15**	+	+	-	+	-	*P. koreensis*	LR027419
	17		**2.SG.16**	+	+	-	+	-	*P. koreensis*	LR027420
	18		**2.SG.17**	+	+	-	+	-	*P. koreensis*	LR027421
	19		**2.SG.18**	+	+	+	+	-	*P. koreensis*	LR027422
	20		**2.SG.20**	+	+	+	+	-	*P. koreensis*	LR027423
	21	**125**	**3.SG.1**	+	+	+	+	-	*P. fluorescens*	LR027430
	22		**3.SG.1°**	+	+	+	+	-	*P. fluorescens*	LR027431
	23		**3.SG.2°**	+	+	+	+	-	*P. koreensis*	LR027432
	24		**3.SG.9**	+	+	+	+	-	*P. koreensis*	LR027433
	25		**3.SG.13**	+	+	+	+	-	*P. moraviensis*	LR027434
	26		**3.SG.15**	+	+	+	+	-	*P. koreensis*	LR027435
	27		**3.SG.19**	+	+	+	+	+	*P. fluorescens*	LR027436
	28		**3.SG.32**	+	+	+	+	-	*P. gessardii*	LR027437
	29	**147**	**4.SG.1**	+	+	+	-	-	*P. moraviensis*	LR027443
	30		**4.SG.2**	-	-	-	-	-	*Microbacterium oxydans*	LR027444
	31		**4.SG.3**	+	+	-	+	-	*P. koreensis*	LR027445
	32		**4.SG.4**	+	+	-	-	-	*P. cedrina*	LR027446
	33		**4.SG.5**	+	+	+	-	-	*P. koreensis*	LR027447
	34		**4.SG.6**	+	+	+	-	-	*P. koreensis*	LR027448
	35		**4.SG.6S**	-	+	+	-	-	*P. koreensis*	LR027449
	36	**188**	**5.SG.2**	-	-	+	+	+	*P. thivervalensis*	LR027455
	37		**5.SG.3**	+	+	+	-	+	*Bacillus amyloliquefaciens*	LR027456

	38		**5.SG.6**	-	+	-	-	-	*Paenibacillus amylolyticus*	LR027457
	39		**5.SG.9**	+	+	-	-	-	*P. moravensis*	LR027458
	40		**5.SG.10**	-	-	-	-	+	*Paenibacillus polymyxa*	LR027459
	41		**5.SG.11**	-	-	+	-	-	*Cellulosimicrobium funkei*	LR027460

DAS: days after sowing; **P**: phosphate solubilization; **Fe**: siderophore production; **U**: ureolytic activity. **Fluo**: Isolates with fluorescent pigment production after growing on King medium B and inspecting under UV light.
